# High-intensity ultrasound modified the functional properties of *Neosalanx taihuensis* myofibrillar protein and improved its emulsion stability

**DOI:** 10.1016/j.ultsonch.2023.106458

**Published:** 2023-05-27

**Authors:** Xiao-hong Deng, Xiang-xiang Ni, Jia-hui Han, Wen-hua Yao, Ya-jie Fang, Qin Zhu, Ming-feng Xu

**Affiliations:** Key Laboratory for Quality and Safety of Agricultural Products of Hangzhou City, College of Life and Environmental Sciences, Hangzhou Normal University, Hangzhou, Zhejiang 311121, China

**Keywords:** *Neosalanx taihuensis*, Ultrasound, Myofibrillar protein, Functional properties, Emulsion stability

## Abstract

This study aimed to investigate the effects of high-intensity ultrasound treatment on the functional properties and emulsion stability of *Neosalanx taihuensis* myofibrillar protein (MP). The results showed that the carbonyl groups, emulsification properties, intrinsic fluorescence intensity, and surface hydrophobicity of the ultrasound treated MP solution were increased compared to the MP without ultrasound treatment. The results of secondary structure showed that the ultrasound treatment could cause a huge increase of β-sheet and a decline of α-helix of MP, indicating that ultrasound induced molecular unfolding and stretching. Moreover, ultrasound reduced the content of total sulfhydryl and led to a certain degree of MP cross-linking. The microscopic morphology of MP emulsion indicated that the emulsion droplet decreased with the increase of ultrasound power. In addition, ultrasound could also increase the storage modulus of the MP emulsion. The results for the lipid oxidation products indicated that ultrasound significantly improved the oxidative stability of *N. taihuensis* MP emulsions. This study offers an important reference theoretically for the ultrasound modification of aquatic proteins and the future development of *N. taihuensis* deep-processed products represented by surimi.

## Highlights

• High-intensity ultrasound caused oxidation and aggregation of *N. taihuensis* MP.
• High-intensity ultrasound improved functional properties of *N. taihuensis* MP.
• High-intensity ultrasound enhanced viscoelasticity of *N. taihuensis* MP emulsion.
• High-intensity ultrasound improved oxidative stability of *N. taihuensis* MP emulsion.

## 1 Introduction

*Neosalanx taihuensis* belongs to the order *Scleractinia*, *Salmoniformes*, which is a high-protein and low-fat food and rich in thiamine, riboflavin, nicotinic acid and other nutrients. *N. taihuensis* is tender and edible as a whole, and is now recognized by the international nutrition community as a natural “longevity food”. China is the origin and main distribution area of *N. taihuensis* in the world. 15 of the world's 17 species of *N. taihuensis* are distributed in the eastern China offshore including the Yangtze River basin [1]. However, there are few deep-processed *N. taihuensis* products, so it is of great economic value to develop high-value-added functional protein products. Myofibrillar protein (MP) accounts for approximately more than 50% of the total *N. taihuensis* protein which has a good gel-forming ability and can also be used as a raw material for surimi-based products. MP contributes to more than 90% of the development of the emulsifying capability of aquatic muscle food. It could be adsorbed to form an interfacial protein membrane on the surface of fat globules/oil droplets in the emulsified meat system [2], [3], [4]. In addition, MPs are easily digestible and rich in all essential amino acids, which make them popular functional ingredients in food formulations and allowing the preparation of stable emulsions as nutrient delivery systems. In order to develop high-quality *N. taihuensis* muscle food and related new nutritional products, it is necessary to investigate the correlation between the modification of the structural and physicochemical properties of *N. taihuensis* MP and the improvement of its functional properties [5].

Currently, scholars have mainly studied the modification of MP in the following four main approaches: a) Physical methods such as heating, freezing and high pressure; b) Chemical methods such as acid method, alkali method and glycosylation; c) Bioengineering techniques by enzymatic and microbiological methods; d) New technologies such as ultrasound and radiation [6]. Among them, ultrasound technology has a wide range of applications in the functional properties, emulsification, defoaming, and microstructural modification of aquatic proteins due to its advantages of high efficiency, easy control and simple operation [7]. Amiri et al. [8] found that ultrasound treatment could improve the rheological properties by modifying its functional and physicochemical properties. Li et al. [9] showed that the MP emulsification behavior was significantly improved after ultrasound, and a more stable emulsion could be obtained from the MP. Similarly, Li et al. [2] also reported that ultrasound treatment improved the functional properties of shrimp MP and its gel properties. At present, there are few literatures on the effects of processing technology on the *N. taihuensis* MP and its related surimi products, mainly focusing on squid, shrimp and poultry meat.

Therefore, the aim of this work was to investigate the effects of ultrasound treatment (power 200, 400 and 600 W, 20 kHz, 60 min) on the functional properties and emulsion stability of *N. taihuensis* MP. Carbonyl groups, emulsifying activity index (EAI), emulsifying stability index (ESI), sulfhydryl groups, surface hydrophobicity, intrinsic fluorescence spectrum, circular dichroism (CD) spectrum, subunit proteins and aggregation were determined to reflect changes in the structural and functional properties of MP. In addition, microscopic morphology, particle size distribution, rheological properties, and TBARS were determined to reflect changes in the emulsion functional properties of MP and the storage stability of the emulsion. This study offers an important reference theoretically for the industrial production of protein-based foods using ultrasound treatment and the deep processing of *N. taihuensis* into surimi gel products.

## 2 Materials and methods

### 2.1 Materials

Frozen *N. taihuensis* with average weight and length of 8.52 ± 0.60 g and 9.33 ± 0.32 cm were purchased from Taihu Fishery Market (Huzhou, China) in September 2022. Yu-Hang Jiang, a professor from Zhejiang Aquatic Products Research Center, identifed the varieties of purchased *N. taihuensis*. 5,5′-dithiobis (2-nitrobenzoic acid, DTNB, BC Grade, CAS: 69–78-3) and 1,4-piperazine bis (ethanesulfonic acid, PIPES, Molecular Biology Grade, purity ≥ 99%, CAS: 5625–37-6) were purchased from Zhijiang Yunrui Huaxue Co., Ltd. (Hangzhou, China). DL-Dithiothreitol (DTT, Molecular Biology Grade, purity ≥ 99%, CAS: 3483–12-3) and 2, 4-dinitrophenylhydrazine (DNPH, Molecular Biology Grade, purity ≥ 97%, CAS: 119–26-6) were purchased from Hangzhou Jurui Reagent Co., Ltd. (Hangzhou, China). Except for the above reagents, all other reagents used in the experiments were purchased from Damao Reagent Company (Tianjin, China) of analytical grade.

### 2.2 Preparation of *N. Taihuensis* MP

The *N. taihuensis* MP was extracted with a reference of Li et al. [10] using a homogenizer (model T25, IKA Corporation, Staufen, Baden-Wurttemberg, Germany) at 10000 rpm for 2 min. The extraction buffer (pH 7.0) consisted of 0.1 M NaCl, 1 mM EDTA, 2 mM MgCl_2_, and 0.1 M Na_2_HPO_4_/NaH_2_PO_4_. The *N. taihuensis* meat to liquid ratio was 1:4 (w/v). After homogenization, the above mixture was centrifuged three times using a Hitachi centrifuge (CF16RN, Tokyo, Japan) at 10,000 g and 4 °C for 15 min. The final precipitate obtained was the crude *N. taihuensis* MP. The MP was stored at 4 ℃ and used up within 48 h.

### 2.3 Ultrasound treatment

The ultrasound treatment of *N. taihuensis* MP was performed with a reference of our previous method [2]. An ultrasonic cell disruptor (SCIENTZ-II D, Ningbo, China) was used to treat MP solution. Under ice bath conditions, the titanium probe (diameter, 6 mm) was inserted into the protein solution. The titanium probe was stretched into the *N. taihuensis* MP solution (25 mg/mL) under the circulating ice-cold bath condition with a depth of 2.5 cm. The ultrasound power was set at 0, 200, 400 and 600 W, respectively, and the ultrasound treatment was performed for 60 min at an amplitude and frequency of 60% and 20 Hz, respectively. MP without ultrasound treatment was used as blank control.

### 2.4 Carbonyl content

With reference to the method of Li et al. [10], the carbonyl group content of the *N. taihuensis* MP (5 mg/mL) was determined using a protein carbonyl test kit purchased from Nanjing JIANCHENG Biology Co., Ltd. (Item A087, Nanjing, China). The principle of the carbonyl kit was that the carbonyl group in MP could react with 2,4-dinitrophenylhydrazine to produce red 2,4-dinitrophenylhydrazone. The carbonyl content was expressed as nmol/mg.

### 2.5 Emulsification performance

The determination of emulsification performance of MP including ESI and EAI were practiced with a reference of Li, et al. [9]. Briefly, the soybean oil and MP solutions (10 mg/mL) were mixed (1: 4, w/v) and homogenized twice for 1 min at 10,000 rpm using a blender (T25, IKA Corporation, Staufen, Baden-Wurttemberg, Germany). Then, a SDS solution (1 mg/mL) was used to dilute 50 μL of the emulsion 100-fold. The absorbance at 500 nm of the diluted emulsion was recorded immediately (0 min) and 10 min later, respectively, using a microplate reader (Infinite M200, TECAN, Switzerland).

### 2.6 Tertiary structures

#### 2.6.1 Total sulfhydryl groups

The sulfhydryl group content of *N. taihuensis* MP was determined according to a previous method [10]. In brief, 7.5 mg of MP was mixed with 9.5 mL of Tris-glycine buffer (pH = 8.0, 86 mM Tris, 90 mM glycine) and 0.5 mL of 10 mM DTNB. Then, the above mixture was incubated for 1 h at 25 °C and subsequently centrifuged at 12,000 g for 10 min. The absorbance of the supernatant was measured at 412 nm (*A*_1_). Buffer solution replaced MP solution as blank control (*A*_2_). The total sulfhydryl group was calculated as the following formula: TSG (μmol/g) = (*A*_1_ - *A*_2_) × 14.706.

#### 2.6.2 Surface hydrophobicity

With reference to the method of Chelh, Gatellier, and Santé-Lhoutellier [11], the surface hydrophobicity of the *N. taihuensis* MP (5 mg/mL) was determined using a bromophenol blue solution (1 mg/mL). The solution without MP was used as a blank. The samples and control were shaken for 10 min at 25 ℃, and then centrifuged for 15 min at 2000 g. The amount of bromophenol blue binding was calculated by the absorbance value at 595 nm for a microplate reader (Infinite M200, TECAN, Mannedorf, Switzerland) to express the hydrophobicity intensity of the MP surface.

#### 2.6.3 Intrinsic fluorescence spectrum

A fluorescence spectrophotometer (RF-6000, Shimadzu, Tokyo, Japan) was used to measure the endogenous fluorescence spectrum of *N. taihuensis* MP. Firstly, the MP sample was diluted to 0.2 mg/mL with a phosphate buffer containing 0.6 M NaCl. The emission wavelength was set from 300 to 400 nm and the excitation wavelength was set at 280 nm with a scanning speed of 1 nm/s [10].

### 2.7 Secondary structures

#### 2.7.1 Fourier transform infrared spectrum

The lyophilized powder of *N. taihuensis* MP (1.2 mg) was mixed with 100 mg of KBr powder in an agate mortar and recorded in a range of 4000 to 400 cm^−1^ at 25 ± 1 ℃ using a FTIR (Perkin Elmer, Massachusetts, USA) [12].

#### 2.7.2 Circular dichroism spectrum

The quantitative analysis of *N. taihuensis* MP secondary structure was carried out by a CD spectrometer (J-1500, JASCO, Tokyo, Japan) according to the method of Yan et al. [12]. The parameters of CD spectrum were set as follows: wavelength range, 200–260 nm; scanning speed, 200 nm/min; bandwidth, 2.0 nm.

### 2.8 SDS-PAGe

Sodium dodecyl sulfate–polyacrylamide gel electrophoresis (SDS-PAGE) was used to check the crosslinking of the MP sample. Before sample loading, 1 mL of MP sample and a 2.7 mL of 5% SDS solution were mixed, then heated at 80 °C for 1 h and centrifuged (25 ℃) at 3000 g for 15 min. The supernatant was then diluted to a protein concentration of 4 mg/mL. A slab gel system including a 5% polyacrylamide stacking gel and a 10% polyacrylamide resolving gel, and each well was loaded with 30 μg of MP [13]. The gel sheets were placed in Coomassie brilliant blue solution R250 for 5 h and then decolored on a shaker overnight with a decolorization solution containing 50% anhydrous ethanol and 9% glacial acetic acid. Gels images were observed by a DNR Bio-Imaging systems.

### 2.9 Preparation of the MP stabilized emulsion

The MP stabilized emulsion was prepared at 25 ± 2 ℃ by homogenizing the *N. taihuensis* MP suspension (20 mg/mL) and oil (25%, w/w) using a homogenizer (T25, IKA Corporation, Staufen, Baden-Wurttemberg, Germany) at 17,500 rpm for 3 min. After that, the above obtained emulsion was immediately integrated with MP suspensions (40 mg/mL protein) in a 50 mM PIPES buffer (0.6 M NaCl, pH 6.25) to obtain a MP stabilized emulsion (30 mg/mL MP and 10% lipid) [14].

### 2.10 Morphological examination

Briefly, imaging slides for each sample were prepared, 10 μL emulsion was applied, and the slide was coated with a cover glass for the microscope. An inverted microscope was then used to observe the samples' morphology in a bright field (ECLIPSE Ti-S, Nikon, Tokyo, Japan).

### 2.11 Particle size distribution

The particle size distribution of emulsions was carried out according to the method of Ma et al. [15], [16]. An acoustic spectrometer (DT-1202, Dispersion Technology, California, USA) was used to determine the particle size distribution of the emulsions by ultrasound means. The calculation was based on the software provided by the manufacturer (Malvern Ltd.).

### 2.12 Rheological properties

The dynamic viscoelasticity of the MP emulsion was determined according to the method of Cao and Xiong [17] at a frequency of 10 rad / s and a strain of 1%. The temperature rise procedure was as follows: 20–72 °C, 1 °C / min. The storage modulus (G′) was recorded during dynamic rheological measurements.

### 2.13 Oxidative stability

The emulsions could be kept in a refrigerator for up to 6 days at 4 °C. On days 0, 3, and 6, the amount of 2-thiobarbituric acid-reactive substances (TBARS) produced was measured to determine lipid oxidation products during storage [14]. The TBARS of the emulsions was determined referring to Zhao et al. [18] by mixing 0.3 mL of emulsion and 0.7 mL of deionized water with 2 mL of TBA reagent in 15 mL test tubes.

### 2.14 Statistical analysis

All the above experiments were at least three independent experimental replicates. The results of this study were compared using one-way analysis of variance followed by the Student-Newman-Keuls model in the case of significant differences (*p* < 0.05), using SPSS 19.0 (IBM Inc., Chicago, USA). The graphs in the document were completed through Origin 2018 (OriginLab Inc., Northampton, USA).

## 3 Results and discussion

### 3.1 Carbonyl of MP

**Fig. 1:**
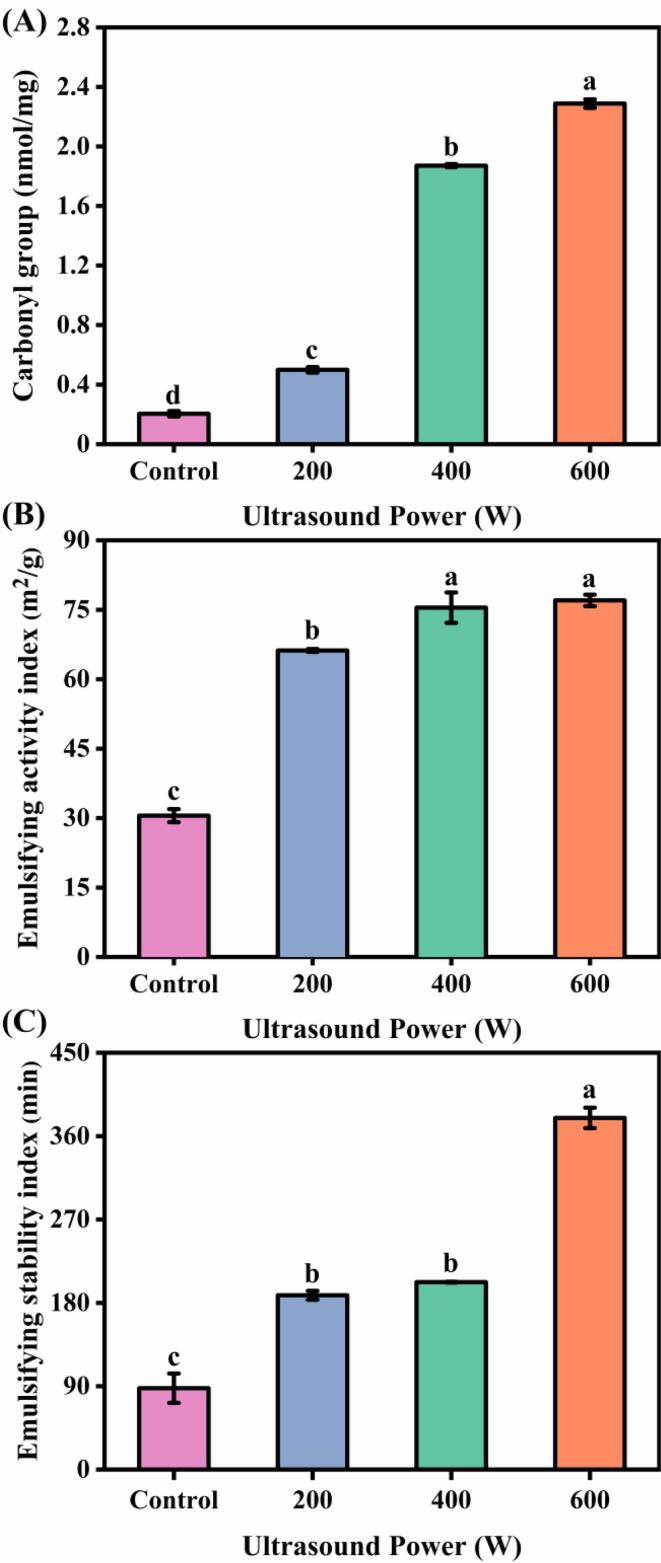
Effect of different ultrasound power on the carbonyl (A), emulsifying activity index (B) and emulsifying stability index (C) of *N. taihuensis* MP. a-d: Different letters above standard deviation bar indicate significant differences among the means (*p* < 0.05).

The side chains of amino acid residues such as lysine, threonine, proline and arginine in proteins are easily oxidized by the action of various reactive oxygen groups to form carbonyl groups. The content of carbonyl groups is a common indicator of protein oxidation [19]. The effect of ultrasonic power on the carbonyl content of *N. taihuensis* MP is shown in Fig. 1A. The carbonyl content of *N. taihuensis* MP without ultrasound treatment was 0.20 nmol/mg, which increased significantly after ultrasound treatment. It indicated that ultrasound treatment could modify the molecules and induce a certain level of oxidation of *N. taihuensis* MP, and this oxidation becomes more pronounced with higher ultrasonic power. For example, the carbonyl content reached a maximum (*p* < 0.05) when the ultrasound power was increased to 600 W, which was approximately 11.45 times higher than that of the control group. Generally speaking, the carbonylation of proteins is mainly attributed to the oxidation of amino acid side chains and the aldehydes produced by lipid oxidation can also bind to amino acid side chain groups to form Schiff bases thereby increasing the carbonyl content [2]. In this study, ultrasound led to the increase of carbonyl groups in *N. taihuensis* MP, which might be due to the fact that the secondary structure of *N. taihuensis* MP contained many loose and disordered random curl structures. Ultrasound-induced cavitation can generate localized high temperatures, resulting in the conversion of reducing amino acids in protein into carbonyl groups through oxidation [20].

### 3.2 EAI and ESI of MP

The MP molecule has hydrophobic and hydrophilic groups, which allow it to act as an emulsifier in the oil and water phases, respectively [21]. Thermal aggregation affects the molecular structure of MP, which in turn affects its emulsifying properties. As shown in Fig. 1B-C, the EAI and ESI of *N. taihuensis* MP without ultrasound treatment were 30.53 m^2^/g and 87.84 min, respectively, which increased significantly after ultrasound treatment. For example, ultrasound power from 0 to 600 W caused an increase in MP emulsification performance, including an increase of 1.52-fold in EAI and an increase of 3.32-fold in ESI (*p* < 0.05). Usually, EAI indicates the ability of MP to form an emulsion, while ESI is used to indicate the ability of MP to form a stable emulsion over a period of time. Emulsification properties are important functional properties of MP, which play an important role in MP-related new products development [9]. Li et al. [2] also reported that the emulsification characteristics of *Penaeus vannamei* MP increased significantly after 500 W ultrasonic treatment, which was highly consistent with the results of this study. It was suspected that ultrasonic power might increase the specific surface area of MP and its intermolecular electrostatic repulsion and hydrophobic interaction, thereby improving the stability of protein solution and the ability of lotion interface to absorb protein, and ultimately enhancing EAI and ESI.

### 3.3 Tertiary conformations of MP

**Fig. 2:**
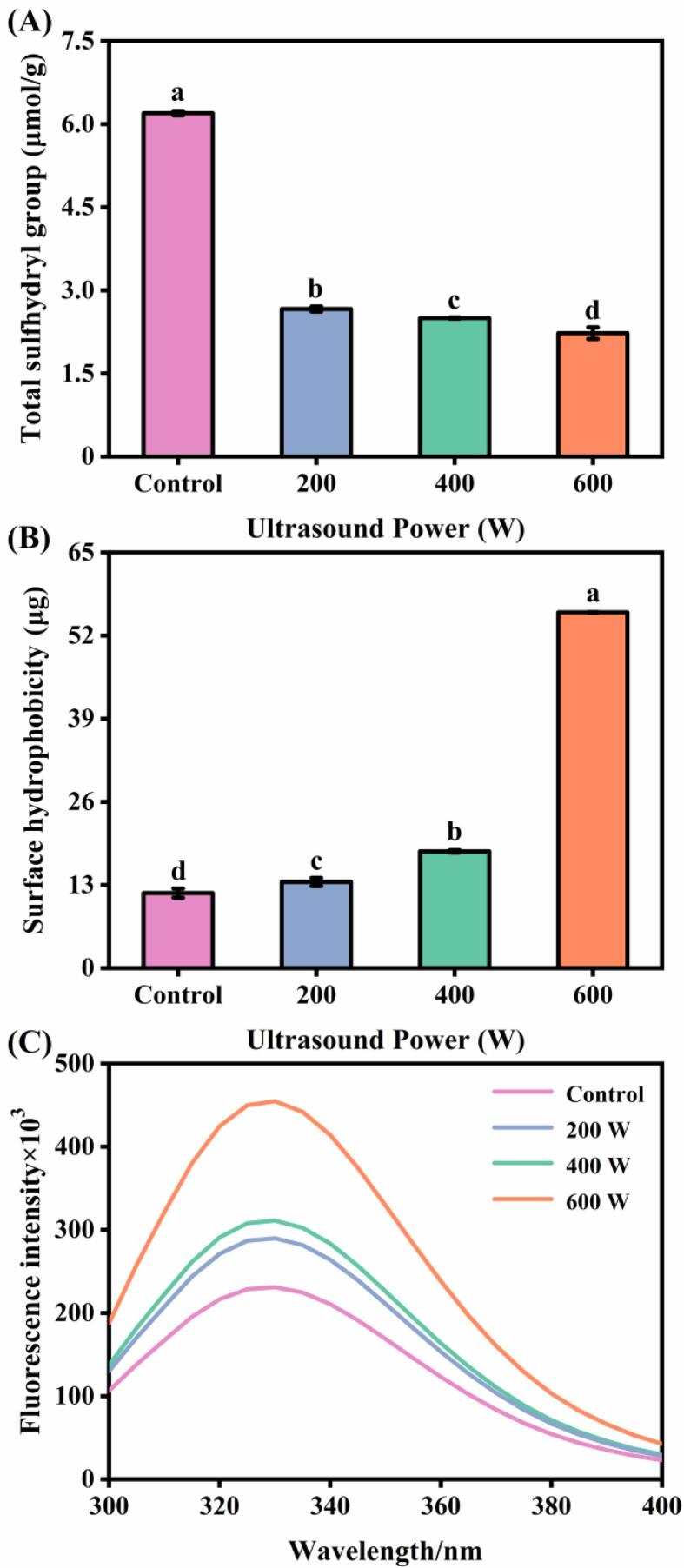
Effect of different ultrasound power on the total sulfhydryl content (A), surface hydrophobicity (B) and intrinsic fluorescence intensity (C) of *N. taihuensis* MP. a-d: Different letters above standard deviation bar indicate significant differences among the means (*p* < 0.05).

The sulfhydryl group of cysteine is very reactive and highly susceptible to disulfide bond formation by free radical attack, so the sulfhydryl content can also be used to react to the degree of protein oxidation. Myosin, as the main component of MP, contains sulfhydryl groups that are easily attacked by free radicals and easily form intramolecular and intermolecular disulfide bonds [22]. As shown in Fig. 2A, ultrasound treatment from 0 to 600 W caused a decrease (approximately 64.04%, *p* < 0.05) in total sulfhydryl group of MP. This might be because the hydrogen atom in the sulfhydryl group was captured by the free radicals generated in the ultrasound treatment process, resulting in the oxidation of the sulfhydryl group and the formation of disulfide bonds within or between the peptides [23]. Li et al. [2] also found that the total sulfhydryl group of shrimp MP declined with increasing power of ultrasound.

Surface hydrophobicity reflects the changes in the number and distribution of hydrophobic groups on the protein surface, which can be characterized by the binding strength of bromophenol blue to the protein [24]. As shown in Fig. 2B, the surface hydrophobicity of *N. taihuensis* MP leveled up obviously from 11.74 μg (0 W) to 55.63 μg (600 W) after ultrasound treatment, an increase of approximately 3.74-fold (*p* < 0.05). This might be due to the mechanical force caused by the cavitation effect of ultrasound that disrupted the intermolecular forces of MP, allowing the structure of MP to unfold and thus exposing more buried hydrophobic groups inside [25]. This result was in consistent with the finding of Chen et al. [26], who reported that proteins undergo unfolding and complexation during sonication, accelerating the exposure of nonpolar and hydrophobic amino acids (methionine, phenylalanine, tryptophan, etc.) inside the molecule, thus increasing their surface hydrophobicity.

The change in conformation of the MP tertiary structure can be determined by the change in the maximum absorption wavelength of the intrinsic fluorescence. Fig. 2C shows the intrinsic fluorescence spectrum of MP with different ultrasound treatments (200, 400, and 600 W), which reflects the changes of tryptophan residues as part of the tertiary conformation of MP. The fluorescence intensity of *N. taihuensis* MP increased significantly from 231.15 × 10^3^ (0 W) to 454.99 × 10^3^ (600 W) after ultrasound treatment, which was linearly related to the change in surface hydrophobicity. It was hypothesized that when the α-helical structure of MP was transformed to a randomly coiled structure, the molecular structure became loose and the hydrophobic regions encapsulated in the interior outflip to the surface of the protein molecule, resulting in an increase in fluorescence intensity [16]. Alternatively, it was also possible that the fluorescence intensity was increased due to the aggregation of the protein interior of MP under ultrasound conditions, generating macromolecular material.

### 3.4 Secondary structures of MP

**Fig. 3:**
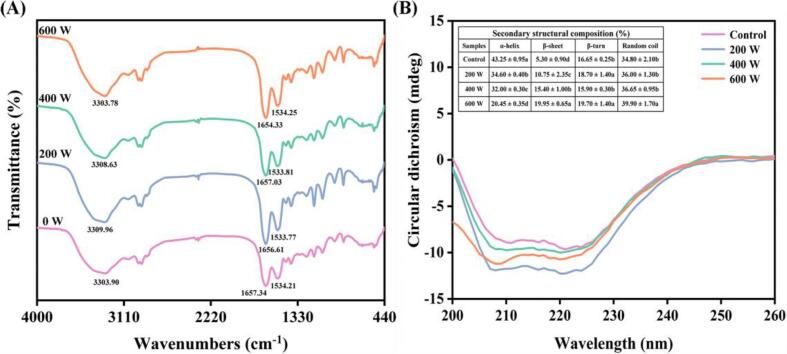
Effect of different ultrasound power on the infrared spectrum (A) and circular dichroism spectrum (B) of *N. taihuensis* MP. a-d: Different letters on each column of data indicate significant differences among the means (*p* < 0.05).

FTIR is a highly sensitive method for examining conformational variations in the secondary structures of proteins. The secondary structures of MP consist of β-sheet, β-turn, α-helix, and random coil, which are intimately linked to its emulsifying characteristics [9]. The FTIR spectra of MP subjected to various ultrasound treatments are presented in Fig. 3A. The spectrum of the control group MP (non-ultrasonic treatment group) displayed three prominent peaks at 1534.25, 1654.33, and 3303.78 cm^−1^, which correspond to the vibrations for the amide Ⅱ, amide I, and amide A of MP, respectively [27]. It is well known that the amide I band (1600–1680 cm^−1^) represents the vibration of the C = O stretching of the peptide bond, which corresponds to different secondary structures [28]. The results of this study showed that the effects of different ultrasound power on the secondary structures of MP were different due to the difference in wavenumber shifts.

In order to further quantify the effect of different ultrasonic treatments on the secondary structure of protein, CD spectra was used to obtain more quantitative data about the composition of secondary structure. The relative contents of α-helix, β-sheet, β-turn and random coil structures of MP are shown in Fig. 3B. Nearly 50% of secondary structures were based on α-helix and β-sheet, which are the elastic structures of proteins. β-turn was one of the indicators reflecting the looseness of protein structure, and random curl played an important role in MP conformation. In this study, 43.25% α-helix and 5.30% β-sheet were detected in MP without ultrasound treatment. After ultrasound power from 0 to 600 W, the α-helix significantly decreased by 52.72%, but the β-sheet increased by a 2.76-fold (*p* < 0.05). The above results show that the secondary structure of the MP unfolded in a deconvoluted helical direction after ultrasonication. The α-helix structure of MP is primarily stabilized by the hydrogen bond between the amino hydrogen and the carbonyl oxygen of the polypeptide chain, and the cavitation effect of ultrasound changed the internal spatial structure of the MP molecules, leading to a decrease in α-helix. The observed increase in β-sheet content could be attributed to the reduction in α-helix content [29]. Li et al. [2] also reported found that the content of α-helix descended by 29.90% but β-sheet went up by a 4.36-fold after ultrasound treatment. This was in complete accord with the results of this study.

### 3.5 SDS-PAGE of MP

**Fig. 4:**
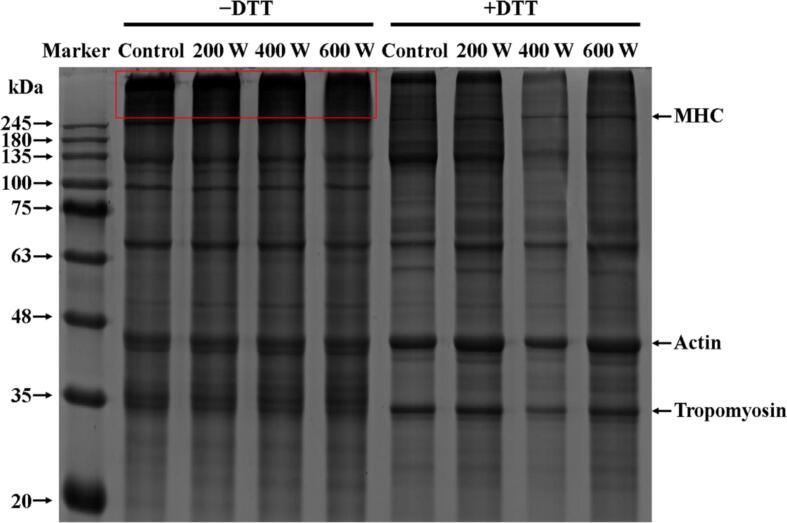
Effect of different ultrasound power on the protein composition of *N. taihuensis* MP.

To study the role that polymers played during protein gelation, SDS-PAGE was used to analyze the crosslinking of proteins through covalent bonds. In this study, the bands of the major protein molecules of MP had not been altered by ultrasonic treatment in the non-reductive electrophoresis profiles (−DTT). Nonetheless, the presence of significant protein deposition and aggregation at the top of the electrophoresis lane provided evidence that the ultrasonic power treatment resulted in some degree of oxidation and aggregation of MP (Fig. 4). Myofibrin heavy chain (MHC) and actin were the main subunit proteins of MP. The clear MHC and actin bands were found in the reduction electrophoresis map instead of the non-reduction map, which further proved the fact that ultrasound caused the oxidative aggregation of MP. Li et al. [2] also reached a conclusion that there was no obvious change in electrophoretic bands of shrimp MP after ultrasound treatment from 100 to 500 W. This was consistent with the results of this study.

### 3.6 Morphological examination of emulsion

**Fig. 5:**
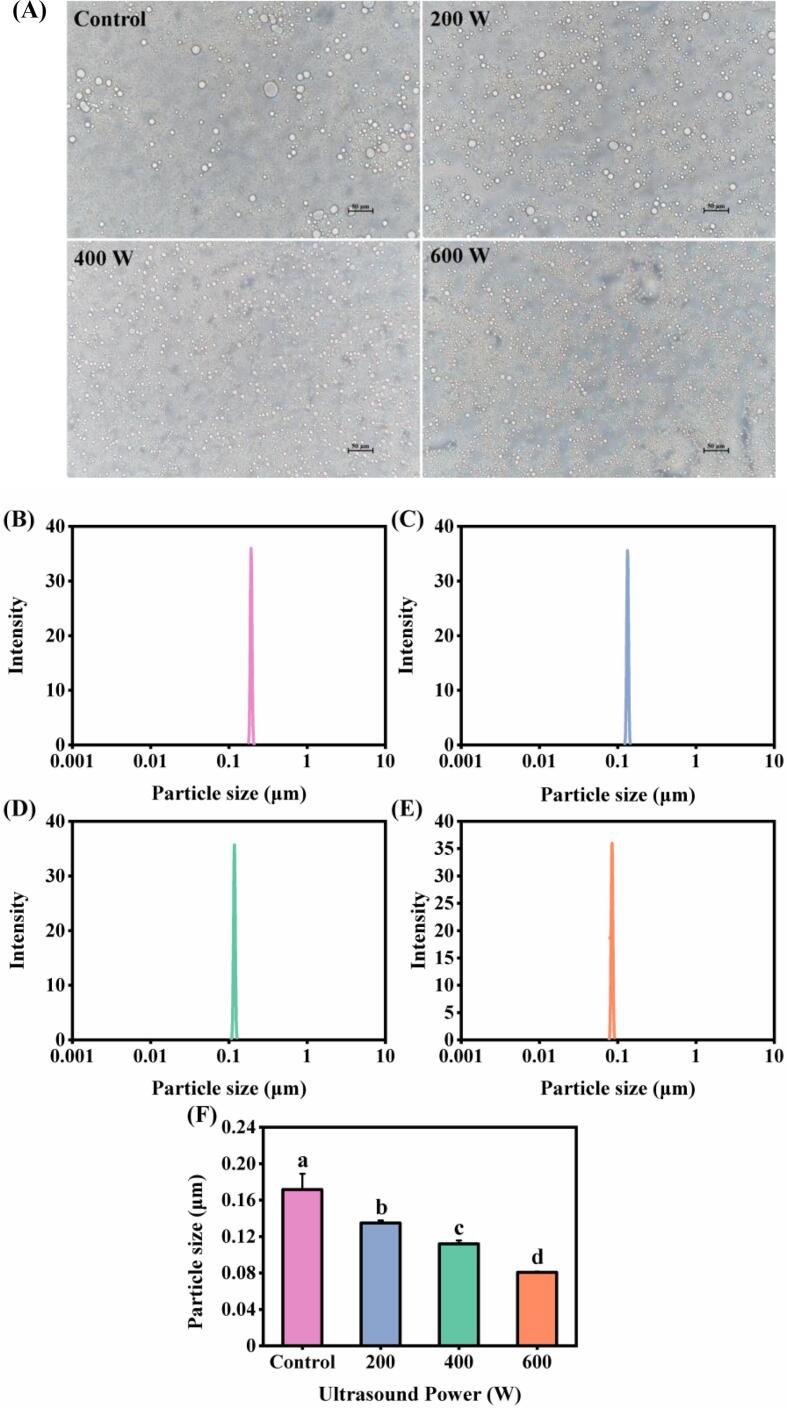
Effect of different ultrasound power on the micromorphology (A) and particle size (B) of *N. taihuensis* MP emulsion. a-d: Different letters above standard deviation bar indicate significant differences among the means (*p* < 0.05).

In order to investigate the effect of ultrasound treatment on the microscopic morphology of *N. taihuensis* MP stabilized emulsion, the microstructure was analyzed by fluorescence inverted microscopy in a bright field mode, and the results are shown in Fig. 5A. It was found that higher power ultrasound treated emulsions stabilized by MP could be observed with a more homogeneous microstructure and smaller oil droplet sizes. For the emulsion prepared by *N. taihuensis* MP without ultrasound treatment, flocculation and aggregation of oil droplets were found, which caused partial coalescence of the emulsion. The coalescence of the emulsions prepared from MP after ultrasound treatment was significantly improved and the emulsions stabilized by MP after 600 W ultrasound treatment had a homogeneous microstructure. These results all indicated that a more homogeneous distribution of oil droplets in the emulsions prepared by the ultrasonicated MP, as the increase in the number of proteins adsorbed on the surface of the droplets causes an increase in their surface hydrophobicity (Fig. 2B). Li et al. [9] also found that the EAI and ESI of chicken breast MP increased after ultrasound treatment, which may be due to the formation of smaller droplets in the samples or the higher surface hydrophobicity of the protein for the ultrasonicated samples.

### 3.7 Particle size distribution of emulsion

The particle size distribution can be used to express the degree of aggregation of proteins or emulsion droplets and is an important parameter affecting the stability of emulsions in its production process [30], [31]. Fig. 5B-E shows the particle size distribution of the emulsion prepared by MP without and with ultrasonic treatment. All emulsion samples had only one single peak, illustrating that the MP stabilized emulsion forms a uniform system, which is consistent with the appearance of the emulsion. As shown in Fig. 5F, the average particle size of the emulsion decreased (from 0.17 to 0.08 μm) with the increase of ultrasound power (from 0 to 600 W). The above results showed that MP after ultrasonic treatment promoted the formation of smaller emulsion droplets compared to untreated MP, which corresponded to the smaller oil droplets observed from the microscopic morphology of emulsion (Fig. 5A). The reduction of MP particle size after ultrasonic treatment was conducive to MP adhering to the water oil interface of the emulsion, which in turn increased the absorption of MP on the surface of oil droplets and prevented gravitational separation, flocculation and agglomeration [32]. Similar results were reported by Ma et al. [16], who found that the emulsion stabilized by cod protein with smaller droplet size had higher stability.

### 3.8 Rheological properties of emulsion

**Fig. 6:**
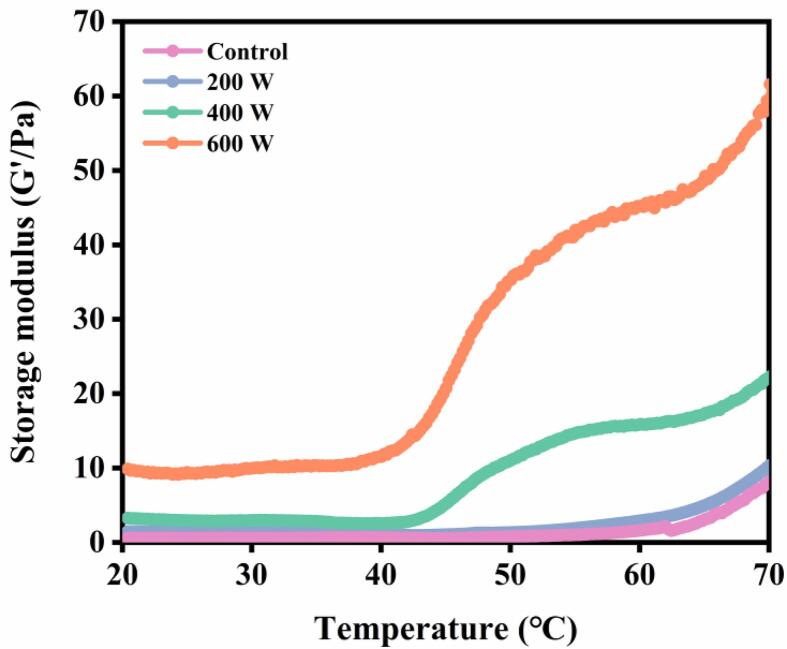
Effect of different ultrasound power on the storage modulus of *N. taihuensis* MP emulsion.

Fig. 6 displays the changes in G′ of MP emulsions following various ultrasound treatments. From 20 to 40 ℃, the gel network gradually formed and G′ steadily increased, which might be caused by the degradation of myosin head [33]. With the continuous rise of temperature, the formation of hydrophobic bonds and S-S in the structure of actin and myosin led to the rapid increase of storage modulus [9]. Overall, ultrasound treatment improved the viscoelasticity of the MP and contributed to gel formation, which in turn led to a power-dependent trend of increasing G′. This might be related to protein chains or protein-coated oil droplets interacting and cross-linking with each other via hydrophobic interactions [34], [35].

### 3.9 Oxidative stability of emulsion

The development and build-up of TBARS, a byproduct of lipid oxidation in emulsion samples, were observed during the storage period of 0 to 6 days at 4 ℃. As shown in Fig. 1S, the concentration of TBARS in the control sample on starting point for storage was approximately 56.11 μM, and the level increased to 149.65 μM (an increase of 1.67-fold, *p* < 0.05) by day 6. In comparison, the TBARS production of the emulsion prepared from the ultrasonically treated MP was significantly lower than that of the emulsion prepared from the MP without ultrasound treatment during the 6 days of storage. For example, in emulsion samples made from the MP treated with 600 W ultrasound, there was less TBARS production (an increase of 1.20-fold by day 6) than in the MP without ultrasound treatment (an increase of 1.67-fold, *p* < 0.05). It was noteworthy that the content of TBARS decreased with increasing ultrasound power at the same storage time. These results suggested that the ultrasonically treated MP could promote binding to oil droplets, reduce the occurrence of lipid oxidation and emulsion breakage, and ultimately improve the storage stability of emulsions. High-intensity sonication might induce more proteins to adsorb to the interface to maintain emulsion stability and formed a spatial site barrier between the aqueous and lipid phases, which provided some degree of inhibition of lipid oxidation [9], [16].

## 4 Conclusion

Ultrasound treatment not only increased the carbonyl, intrinsic fluorescence intensity and surface hydrophobicity of MP, but also changed its secondary structures. These findings suggest that augmenting ultrasonic intensity can enhance both the emulsification efficacy of MP and the endurance of emulsions. Moreover, the application of ultrasound reduced the total sulfhydryl group content in MP, which led to the formation of cross-links and ultimately improved the viscoelastic properties of emulsions. The present study illustrated that ultrasound modified the physicochemical properties and structure characteristics of *N. taihuensis* MP, thereby improving the rheological properties and storage stability of MP stabilized emulsions. This work could provide an important theoretical foundation for utilizing physical techniques in the upgrading process of high-value *N. taihuensis* muscle foods.

## CRediT authorship contribution statement

**Xiao-hong Deng:** Conceptualization, Methodology, Formal analysis, Investigation, Writing – original draft. **Xiang-xiang Ni:** Resources, Investigation. **Jia-hui Han:** Software, Methodology. **Wen-hua Yao:** Methodology, Formal analysis. **Ya-jie Fang:** Methodology, Formal analysis. **Qin Zhu:** Conceptualization, Methodology. **Ming-feng Xu:** Project administration, Supervision, Validation.

## Declaration of Competing Interest

The authors declare the following financial interests/personal relationships which may be considered as potential competing interests: Mingfeng Xu reports financial support was provided by Hangzhou Science and Technology Development Plan.
