# Supplementary material for: High-intensity ultrasound modified the functional properties of Neosalanx taihuensis myofibrillar protein and improved its emulsion stability
Source: Ultrason Sonochem. 2023 May 27;97:106458. doi: 10.1016/j.ultsonch.2023.106458 (PMC10241975; doi:10.1016/j.ultsonch.2023.106458)
Supplement: Supplementary data 1 [file mmc1.docx]

**Fig. 1S**. Effect of different ultrasound power on the TBARS of *N. taihuensis* MP emulsion during storage. a-h: Different letters above standard deviation bar indicate significant differences among the means (*p* < 0.05).
